# Escape of Kdm6a from X chromosome is detrimental to ischemic brains via IRF5 signaling

**DOI:** 10.21203/rs.3.rs-4986866/v1

**Published:** 2024-09-27

**Authors:** Conelius Ngwa, Afzal Misrani, Kanaka Valli Manyam, Yan Xu, Shaohua Qi, Romana Sharmeen, Louise McCullough, Fudong Liu

**Affiliations:** The University of Texas Health Science Center at Houston, McGovern Medical School; The University of Texas Health Science Center at Houston, McGovern Medical School; The University of Texas Health Science Center at Houston, McGovern Medical School; The University of Texas Health Science Center at Houston, McGovern Medical School; The University of Texas Health Science Center at Houston, McGovern Medical School; The University of Texas Health Science Center at Houston, McGovern Medical School; The University of Texas Health Science Center at Houston, McGovern Medical School; The University of Texas Health Science Center at Houston, McGovern Medical School

**Keywords:** Aging, Kdm6a/5c, Microglia, Epigenetics, Ischemia, IRF

## Abstract

The role of chromatin biology and epigenetics in disease progression is gaining increasing recognition. Genes that escape X chromosome inactivation (XCI) can impact neuroinflammation through epigenetic mechanisms. Our prior research has suggested that the X escapee genes *Kdm6a* and *Kdm5c* are involved in microglial activation after stroke in aged mice. However, the underlying mechanisms remain unclear. We hypothesized that *Kdm6a/5c* demethylate H3K27Me3/H3K4Me3 in microglia respectively, and mediate the transcription of interferon regulatory factor 5 (IRF5) and IRF4, leading to microglial pro-inflammatory responses and exacerbated stroke injury. Aged (17–20 months) *Kdm6a/5c* microglial conditional knockout (CKO) female mice (one allele of the gene) were subjected to a 60-min middle cerebral artery occlusion (MCAO). Gene floxed females (two alleles) and males (one allele) were included as controls. Infarct volume and behavioral deficits were quantified 3 days after stroke. Immune responses including microglial activation and infiltration of peripheral leukocytes in the ischemic brain were assessed by flow cytometry. Epigenetic modification of IRF5/4 by *Kdm6a/5c* were analyzed by CUT&RUN assay. The demethylation of H3K27Me3 by kdm6a increased *IRF5* transcription; meanwhile Kdm5c demethylated H3K4Me3 to repress *IRF5*. Both *Kdm6a*^fl/fl^ and *Kdm5c*^fl/fl^ mice had worse stroke outcomes compared to fl/y and CKO mice. Gene floxed females showed more robust expression of CD68 in microglia, elevated brain and plasma levels of IL-1β or TNF-α, after stroke. We concluded that IRF5 signaling plays a critical role in mediating the deleterious effect of *Kdm6a*; whereas *Kdm5c’s* effect is independent of IRF5.

## Introduction

Stroke sensitivity in the aged is driven primarily by sex chromosomes [1, 2], and through a variety of processes including gene epigenetic modifications such as histone methylation [3–5]and demethylation [3, 4]. Histone modifications are indispensable in the regulation of genes for microglia polarization [6–8] and the pathological processes of ischemia [8–11]. In humans, histone modification patterns are associated with X chromosome, especially those genes that escape X-chromosome inactivation (XCI) [12]. Generally, X-linked genes escape XCI at the embryonic stage [13], but it is also suggested that some genes escape with aging [14, 15], leading to gene dosage imbalanced between males and females. Lysine Demethylase 6a (*Kdm6a)* and *Kdm5c*, are escapee genes that encode demethylases of H3K27Me3 [3, 16] and H3K4Me3 [4, 5], respectively. The demethylated form H3K27Me1 is active [17–19] and H3K4Me1 suppressive for gene transcription [19, 20].

Our previous studies have shown that: 1) microglial interferon regulatory factor 5 (IRF5) and IRF4 regulate neuroinflammation in young [21] and aged mice [22]; 2) X chromosomal complement contributes to stroke sensitivity in aged animals [23]; and 3) the X escapee genes *Kdm6a* and *Kdm5c* were involved in IRF5/4 expression and neuroinflammation after stroke [24]. However, the mechanism by which *Kdm6a/Kdm5c* modulates IRF5/4 gene signaling and neuroinflammation after stroke is still elusive. We hypothesized that *Kdm6a/5c* regulate IRF5/4 expression through epigenetic modification of histones, mediate microglial activation/neuroinflammation after stroke, and impact outcomes. To test our hypothesis, we generated microglial *Kdm6a* or *Kdm5c* conditional knockout (CKO) mice, in which one allele of *Kdm6a* or *Kdm5c* is deleted, and subjected the mice to a 60-min middle cerebral artery occlusion (MCAO). Since stroke is a disease that mainly affects the elderly, aged mice (17–20 months) were used in the present study to enhance the translational research potential.

## Materials and methods

### Animal models

*Kdm6a* or *Kdm5c* (CKO) mice were generated by mating *Kdm6a/5c* flox/+ female mice (provided by Dr. Author Arnold, UCLA) with CX3CR1-CreER (strain # 021160, The Jackson Laboratory) males, followed by tamoxifen (TMX) induction [25]. *Kdm6a/5c* CKO female (there is only one allele of the gene in microglia), *Kdm6a/5c* flox/flox (fl/fl; two alleles) female, and *Kdm6a/5c* flox/y (fl/y; one allele) male, aged mice (17–20 months-old) were used in all experiments. All mice were group-housed under pathogen-free conditions with a 12-to-12-h day-night cycle and had access to food and water *ad libitum*. Mice were randomly chosen and used after they were examined free of aberrations or other abnormalities. All studies were conducted in accordance with NIH guidelines for the care and use of laboratory animals and approved by the Institutional Animal Care and Use Committee (IACUC) of the University of Texas Health Science Center at Houston McGovern Medical School.

### Ischemic stroke model

Cerebral ischemia was induced mice by reversible MCAO and under isoflurane anesthesia as previously described [21, 23, 26, 27]. Briefly, a midline ventral neck incision was made, and unilateral MCAO was performed by inserting a 6–0 silicone-coated suture into the right internal carotid artery 6 mm from the internal carotid/ pterygopalatine artery bifurcation via an external carotid artery stump. Reperfusion was performed by withdrawing the suture 60-min after the occlusion. Rectal temperature was maintained at 36.5 ± 0.5°C during surgery with an automated TC-1000 temperature-control feedback system (CWE, Inc., Ardmore, PA, USA). All mice were monitored on a daily basis and then sacrificed at 2 days (for histone mechanistic studies) or 3 days (for stroke outcomes and immune responses) of reperfusion. Sham-operated animals underwent the same procedure including exposure to isoflurane and a midline ventral neck incision, but the suture was not advanced into the MCA. Laser Doppler flowmetry (Moor Instruments Ltd, UK) was applied to measure CBF through the skull at the right temporal fossa. Only the mice whose CBF showed a drop of over 85% of baseline after MCAO was included in the following experiments. The mortality after MCAO was 25% after 2 days stroke and 35% after 3 days stroke. The size of the MCAO-induced infarct was measured by Cresyl violet (CV) staining as described in [23].

### Flow cytometry

Flow cytometry was performed as previously described with modifications [22]. Briefly mice were euthanized and transcardially perfused with 1% heparin in cold PBS, and the brains were harvested. The ipsilateral hemispheres were diced and placed in complete RPMI 1640 (cat # 30–200, ATCC) medium and mechanically and enzymatically digested in collagenase/dispase (1 mg/mL) and DNAse (10 mg/mL) purchased from Roche Diagnostics, for 1 h, and at 37 °C. The cell suspension was diluted in regular RPMI 1640 and then filtered through a 70 μm filter and placed into a 70%/ 30% Percoll gradient. Cells were harvested from the interphase portion of the gradient, washed, and blocked with purified rat anti-mouse CD16/CD32 (mouse BD FC block, cat # 553142) and then stained for extracellular or intracellular markers and using primary antibody-conjugated fluorophores including: anti-Ly6C Brilliant violet 605 (cat # 128035), anti-IL-10 PerCP-Cy5.5 (cat # 505028) purchased from BioLegend. Anti-CD45.2 eF450 (cat # 48–0451-82), anti-CD11b AF488 (cat # 53–0112-82), anti-IL-1β PE (cat # 12–7114-82), anti-Ly6G PE-eFluor 610 (cat # 61–9668-82), anti-TNFα PE-Cy7 (cat # 25–7321-82), anti-IL-4 APC (Cat # 17–7041-82), anti-CD206 AF700 (cat # 56–2061-82), and anti-CD68 APC-eF780 (cat # 47–0681-82) purchased from ThermoFisher Scientific. For live/dead cell discrimination, a fixable viability dye, carboxylic acid succinimidyl ester (CASE-AF350, Invitrogen), was used. Fluorescence minus ones (FMOs) and beads compensations were used for all staining experiments. Data were acquired on Cytoflex_AS41045 (Beckman Coulter) and analyzed using FlowJo (Treestar Inc.).

### mRNA extraction and real-time polymerase chain reaction (RT-PCR)

RT-PCR was performed as in [28] with slight modifications. Briefly total RNA was extracted using RNeasy Mini Kit_74104 (QIAGEN, Germantown, MD, USA) according to the manufacturer’s protocol, and quantified using NANODROP ONE (Thermo Fisher Scientific). The RNA was converted to cDNA by iScript^™^ Reverse Transcription Supermix_1708841. C1000 Touch Thermal Cycler CFX384 Real-Time System (Bio-Rad, Hercules, CA, USA) and the SsoAdvanced Universal SYBR Green Supermix_1725274 (Bio-Rad) were used to perform qPCR. The following gene primers from Integrated DNA Technologies (Coralville, IA, USA) were used: Kdm6a F_ CCAATCCCCGCAGAGCTTACCT, R_TTGCTCGGAGCTGTTCCAAGTG; Kdm5c F_ACCCACCTGGCAAAAACATTGG, R_ACTGTCGAAGGGGGATGCTGTG and the housekeeping gene GAPDH F_GTGTTCCTACCCCCAATGTGT, R_ ATTGTCATACCAGGAAATGAGCTT [24]. The results are reported as normalized fold changes in mRNA, which were determined by the ΔΔCt method using the threshold cycle (Ct) value.

### Neurologic deficit scores (NDS)

Neurological deficits were assessed by the Benderson score system from 0 to 4 as in [21, 22]. Briefly 0-no deficit; 1-forelimb weakness, torso turning to the ipsilateral side when held by the tail; 2-circling to the affected side; 3-unable to bear weight on affected side, and 4-no spontaneous activity or barrel rolling.

### Open field

The open field test (OFT) is a common measure of exploratory behavior, general activity and anxiety-like behavior in rodents, where both the quality and quantity of the activity can be measured [29]. Briefly, mice were placed in a single arena facing the middle of a wall. Mice were allowed to explore the arena for 20 min [23]. After the 20 min duration, the mice were returned to the home cage and arena cleaned with 70% ethanol. The distance moved was analyzed as the locomotor and exploratory behavior of the mice.

### Grip strength

We used the conventional forelimb grip strength test to assess motor function in the mice [30–32]. Briefly, a mouse was gently pulled by its tail ensuring the mouse grips the top portion of the grid and the torso remains horizontal and record the maximal grip strength value of the mouse that is displayed on the screen. This procedure was repeated 3 times to obtain 3 forelimb grip strength measurements for each mouse, and the average strength was calculated.

### Cleavage under targets & release using nuclease (CUT&RUN) assays

IRF DNA binding to histones in microglia was measured by using the CUTANA^™^ ChIC/CUT&RUN Kit (Cat # 14–1048, Epicypher)[33] and CUTANA^™^ DNA purification Kit (Cat # 14–0050)[34], with modifications. Briefly, microglia were isolated for CUT&RUN from mouse brain tissue, using ANTI-PE MICROBEADS (Cat # 130–048-801, Miltenyi Biotech) [35] and PE-TMEM119 monoclonal antibody antibody (Cat # 12–6119-86), binding assays. The ANTI-PE MICROBEADS isolated microglia, were washed with spermidine formulated wash-buffer, and then bound onto activated Concanavalin A conjugated paramagnetic beads (Cat # 21–1401, Epicypher) by gentle agitation for 10 mins and at RT. The cells bound to Concanavalin A beads were then incubated overnight with 2 μg of anti-H3K4Me1 (Cat # 13–0057, Epicypher), anti-H3K4Me3 (Cat # 13–0041, Epicypher), anti-H3K27Me1 (Cat # 61015, Active Motive), or anti-H3K27Me3 (Cat # 13–0055, Epicypher) antibody. After overnight incubation, the antibody-cell-bead complex was washed with wash buffer containing 5% digitonin (permeabilization buffer), and then 3 μL of the cleavage CUTANA^™^ pAG-MNase enzyme (cat# 15–1016, Epicypher)[36], was added and incubated at RT for 10 minutes with no agitation. The complex was further washed with permeabilization buffer and then resuspended in permeabilization buffer (50 μL) and cooled in ice for 4 mins. Digestion, by the pAG-MNase enzyme incorporated into the cells was induced by addition of calcium chloride (100 mM, 1 μL), followed by incubation for 2 hr and at 4 °C. At the end of the digestion step, stop buffer (33 μL, cat # 14–1048, Epicypher) was added to the digestion complex and the complex incubated for 30 mins and at 37 °C without shaking, in order to release DNA fragments. The supernatant containing the released DNA fragments was collected by centrifugation at 16,000 × g, at 4 °C and for 2 min. Total DNA was purified by the CUTANA^™^ DNA purification Kit (Cat # 14–0050) following the vendor’s specifications. DNA was quantified using NANODROP ONE (Thermo Scientific). IRF5 and IRF4 in the purified DNA was quantified by qPCR and using primers including: IRF5 F_5’-GTTTGGTCTGGGTTTTGAGTC-3’, R_5’-ATGTCTGTAACCCTAGCACTTG-3’; IRF4 F_5’-AATGGGAAACTCCGACAGTG-3’, R_5’-TCACGATTGTAGTCCTGCTTG-3’; GAPDH F_5’-GTGTTCCTACCCCCAATGTGT-3’, R_5’-ATTGTCATACCAGGAAATGAGCTT-3’. The results are reported as normalized fold changes in DNA gene expression, which were determined by the ΔΔCt method using the threshold cycle (Ct) value for gene of interest.

### Plasma and brain cytokine levels by conventional enzyme-linked immunosorbent assay (ELISA)

We used the same procedure as in [22, 24] with modification. Briefly blood samples were obtained by cardiac puncture with EDTA-soaked syringed-needles and then centrifuged at 15000 RPM for 20 min, and at 4° C. Brain tissue in non-pyrogenic 5 mL polystyrene round-bottom tubes (Ref # 352235, Corning USA) were homogenized using glass pistons, in complete NP40 buffer, and also centrifuged at 15000 RPM for 20 mins, and at 4 °C. After centrifugation, the supernatant was collected and analyzed with Nunc^™^ MaxiSorp^™^ ELISA plates_423501 and the ELISA MAX^™^ Deluxe kits including TNF_430904, IL-1β_432604, IL-4_431104 and IL-10_431414 (BioLegend USA). Signals were measured at 450 nm in EnSpire^™^ Multimode Plate Reader (Perkin Elmer USA).

### Statistical analysis

Data from individual experiments were presented as mean ± SEM, and assessed by Student’s t test, One-way ANOVA or 2-way ANOVA with Tukey post hoc test for multiple comparisons using GraphPad Prism Software 10.1.2 (324). P < 0.0500 was considered statistically significant. Investigators were blinded to mouse strains for stroke surgery, behavioral testing, infarct, and inflammation analysis.

## Results

### Validation of microglial Kdm6a/Kdm5c CKO mouse model

**Figure 1:**
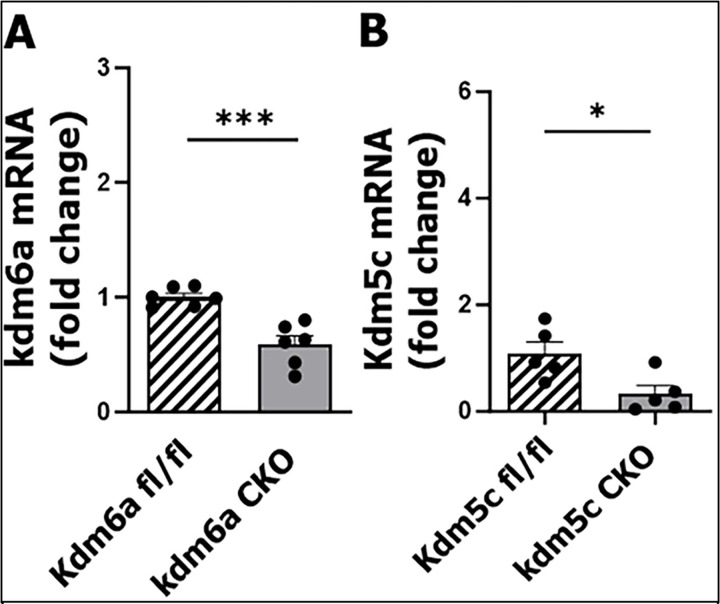
*Kdm6a* and *Kdm5c* mRNA levels in microglia from *Kdm6a*^fl/fl^ vs. CKO (**A**) and Kdm5c^fl/fl^ vs. CKO (**B**) mice. Data was analyzed by Unpaired t Test, n = 5–6 per group; *P < 0.05, ***P < 0.0005.

We generated microglial *Kdm6a* and *Kdm5c* CKO female mouse models, by injecting TMX (75 mg/kg) to *Kdm6a* or *Kdm5c*^fl/+^:CX3CR1-CreER mice (16 months). Four weeks after the TMX injection, the mice were ready for downstream experiments. We validated the CKO mouse model (deletion of one allele of *Kdm6a* or *Kdm5c* in microglia) by isolating microglia (using anti-Tmem119 antibody and ANTI-PE microbeads), followed by q-PCR for *Kdm6a/Kdm5c* mRNA gene expression (fold change) (Fig. 1). The *Kdm6a/Kdm5c* mRNA fold change was significantly reduced in CKO vs. fl/fl microglia (Fig. 1A, 1B), indicating the success of the CKO model.

### Kdm6a modulates IRF5 through H3K27Me3 demethylation

**Figure 2:**
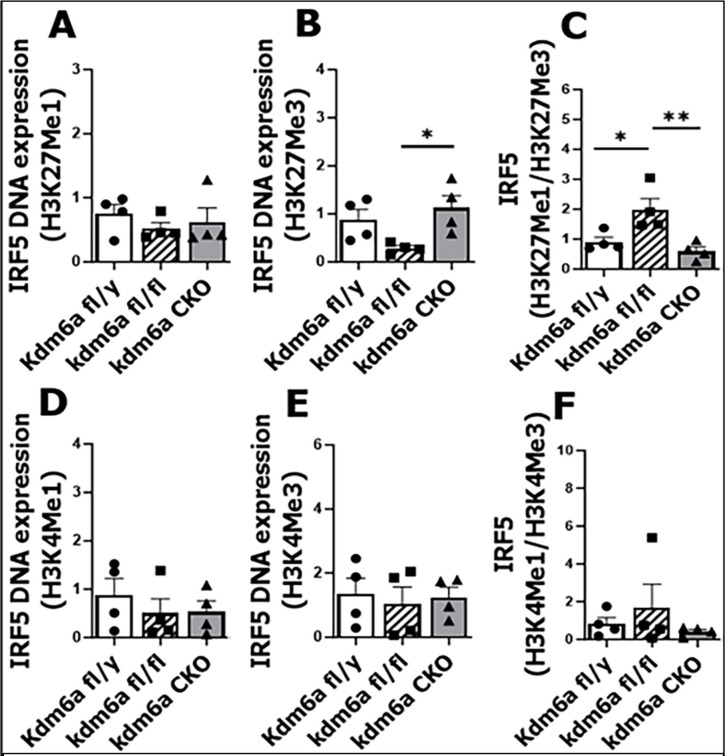
IRF5 DNA binding to histones assayed by CUT&RUN and qPCR after MCAO in *Kdm6a* fl/y, fl/fl, and CKO mice. **(A & B)** IRF5 DNA binding to H3K27Mel and H3K27Me3. (**C**) Ratio of IRF5 DNA binding to H3K27Mel/H3K27Me3. (**D & E**) IRF5 DNA binding to H3K4Mel and H3K4Me3. (**F**) Ratio of IRF5 DNA binding to H3K4Mel/H3K4Me3. n = 4 per group. One Way ANOVA with Tukey’s multiple comparison test. *P < 0.05, **P < 0.005.

Our previous study [24] has suggested that Kdm6a is involved in regulation of IRF5 (pro-inflammatory) signaling. Here we further tested if Kdm6a, a demethylase of histone, can epigenetically modulate IRF5 transcription. We performed MCAO in three strains of mice: *Kdm6a*^fl/y^ (male; one allele of Kdm6a), *Kdm6a*^fl/fl^ (female; two alleles), and *Kdm6a* CKO (female; one allele). At two days after MCAO, microglia were isolated, and CUT&RUN was performed to detect the amount of IRF5 DNA binding to either H3K27Me1(active form for gene expression) or H3K27Me3 (suppressive form). The ratio of IRF5 DNA binding with H3K27Me1 over H3K27Me3 is indicative of the predominance of IRF5 active or suppressive transcription. There was a significant increase in IRF5 DNA binding to H3K27Me3 in *Kdm6a* CKO vs. fl/fl, although no difference was found between strains in H3K27Me1 binding (Fig. 2A&B). The binding ratio of H3K27Me1/H3K27Me3 was significantly higher in microglia isolated from Kdm6a ^fl/fl^ mouse, when compared with either fl/y or CKO (Fig. 2C). There was no significant difference in IRF5 DNA binding to H3K4 between strains (Fig. 2D, E, F). We also examined another transcription factor IRF4 (anti-inflammatory), but found IRF4 DNA binding to either H3K27 or H3K4 did not show any difference between groups (**Suppl. 1 A-F**).

### Kdm5c represses IRF5 transcription through H3K4Me3 demethylation

**Figure 3:**
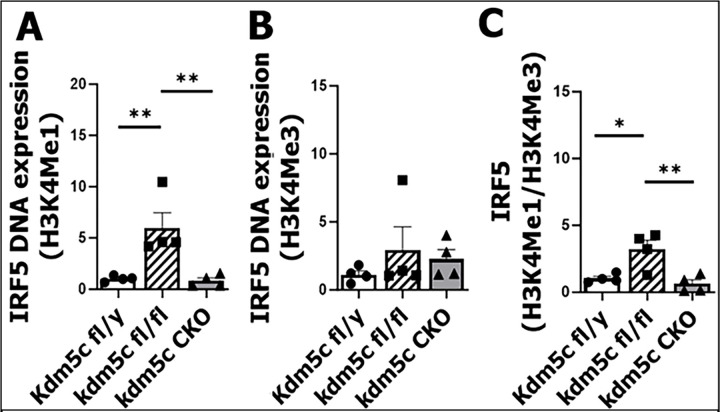
IRF5 DNA binding to histones assayed by CUT&RUN and qPCR after MCAO in *Kdm5c* fl/y, fl/fl, and CKO mice. **(A & B)** IRF5 DNA binding to H3K4Mel and H3K4Me3. (**C**) Ratio of IRF5 DNA binding to H3K4Mel/H3K4Me3. n = 4 per group. One Way ANOVA with Tukey’s multiple comparison test. *P < 0.05, **P ≤ 0.005.

Next, we examined the effect of another histone demethylase/X escapee gene, *Kdm5c*, on IRF5’s transcription with CUT&RUN assay, as *Kdm5c* was also previously found to be involved [24]. The demethylation of microglial H3K4Me3 (active form) by two alleles of *Kdm5c* in *Kdm5c*^fl/fl^ mice induced a significant increase in IRF5 DNA binding to H3K4Me1 form (suppressive) compared with fl/y or CKO mice (both have one allele of *Kdm5c*) (Fig. 3A). The binding ratio of H3K4Me1/H3K4Me3 was significantly higher in the microglia from *Kdm5c*^fl/fl^ vs. fl/y or CKO mice. (Fig. 3A–C). We did not observe any significant difference in IRF5 DNA binding to H3K27 between strains (**Suppl. 2 A-C**). Again, IRF4 DNA binding to either H3K27 or H3K4 did not show difference in these mice (Suppl. **2 D-I).** Taken together, data of Fig. 2&3 suggest that IRF5 transcription is regulated by both Kdm6a and Kdm5c, with an active effect by the former but a suppressive effect by the latter.

### Kdm6a/5c signaling are pro-inflammatory after stroke

**Figure 4:**
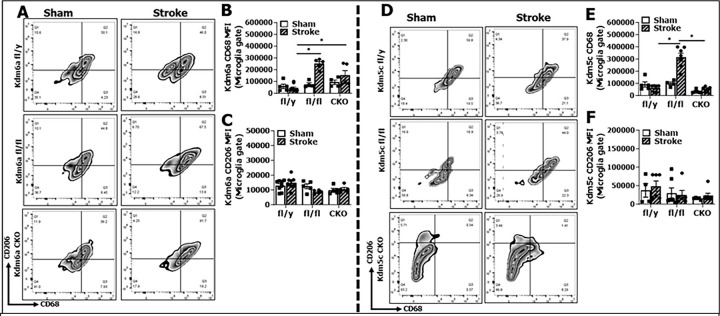
Expression of membrane inflammatory markers on microglia by flow cytometry. (**A**) Representative flow plots of CD68/CD206 expression on microglia from Kdm6a fl/y, fl/fl, and CKO mice. (**B & C**) Mean fluorescence intensity (MFI) of CD68 (B) and CD206 (C) in microglia from sham and stroke mice. (**D**) Representative flow plots of CD68/CD206 expression on microglia from *Kdm5c* fl/y, fl/fl, and CKO mice. (**E & F**) MFI of CD68 (E) and CD206 (F) in microglia from sham and stroke mice, n = 4 per sham and n = 6–8 per stroke group; 2-way ANOVA with Tukey’s multiple comparison test. *P < 0.05.

**Figure 5:**
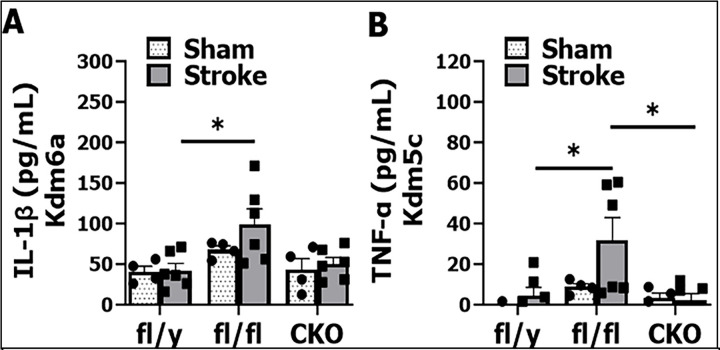
Plasma cytokine levels after stroke. (**A**) IL-1β levels in plasma from Kdm6a fl/y, fl/fl, and CKO mice. (**B**) TNF-α levels in plasma from *Kdm5c* fl/y, fl/fl, and CKO mice, n = 4 per sham and n = 6–8 per stroke group; 2-way ANOVA with Tukey’s multiple comparison test. *p < 0.05.

**Figure 6:**
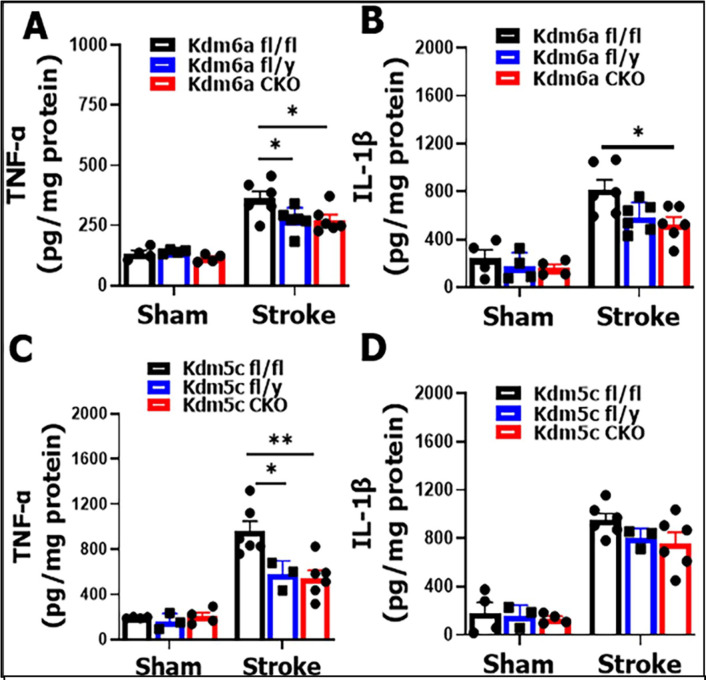
Brain cytokine levels after stroke. (**A & B**) TNF-α and IL-β levels in *Kdm6a* fl/y, fl/fl, and CKO mice brains. (**C & D**) TNF-α and IL-1β levels in *Kdm5c* fl/y, fl/fl, and CKO mice brains, n = 3–4 per sham and n = 3–6 per stroke group; 2-way ANOVA with Tukey’s multiple comparison test. *p < 0.05, **p< 0.005.

Inflammatory responses to stroke can be determined by examination of infiltrating immune cells in the brain, microglial cell membrane and intracellular inflammatory mediator levels, plasma and brain cytokine levels [21, 22, 37]. We first examined membrane and intracellular inflammatory markers in microglia by flow cytometry. The gating strategy for all immune cells is as shown in **Suppl. 3**. CD68 and CD206 are established cell membrane markers for pro- and anti-inflammatory response of microglia respectively [22, 38–40]. In stroke groups, CD68 was significantly increased in the microglia from *Kdm6a*^fl/fl^ vs. fl/y mice, and Kdm5c^fl/fl^ mice had significantly higher CD68 than either fl/y or CKO mice (Fig. 4B, E). However, CD206 expression on microglia did not differ between strains (Fig. 4C, F). We also examined intracellular markers (TNF-α, IL-1β, IL-4, and IL-10) in microglia, but two alleles of *Kdm6a* or *Kdm5c* did not induce higher expression of any of the cytokines compared with one allele of the two genes (**Suppl. 4**). *Kdm6a*^fl/fl^ mice had significantly higher plasma levels of IL-1β than fl/y mice; whereas *Kdm5c*^fl/fl^ mice had higher levels of TNF-α than either fl/y and CKO mice after stroke (Fig. 5A, B). Negative results were found in other cytokines in these mice (**Suppl. 5**). For cytokines assayed in whole brain homogenates, both *Kdm6a*^fl/fl^ and *Kdm5c*^fl/fl^ mice had significantly higher levels of TNF-α than either fl/y or CKO mice after stroke (Fig. 6A, C). Meanwhile *Kdm6a*^fl/fl^ (but not *Kdm5c*^fl/fl^) mice showed a significant increase in IL-1β when compared with CKO (Fig. 6B, D). For anti-inflammatory cytokines, we only found a significant decrease in IL-4 in the *Kdm6a*^fl/fl^ vs. CKO mice brains after stroke (**Suppl. 6A-D**). We also observed significantly greater lymphocyte infiltration in the brains of *Kdm5c*^fl/fl^ mice compared to fl/y or CKO mice after stroke, although there were no significant difference in monocyte or neutrophil infiltration between the strains (**Suppl. 7**). All these data suggest that *Kdm6a* and *Kdm5c* signaling are pro-inflammatory after stroke in the aged.

### Two alleles of Kdm6a exacerbate stroke injury in the aged

**Figure 7:**
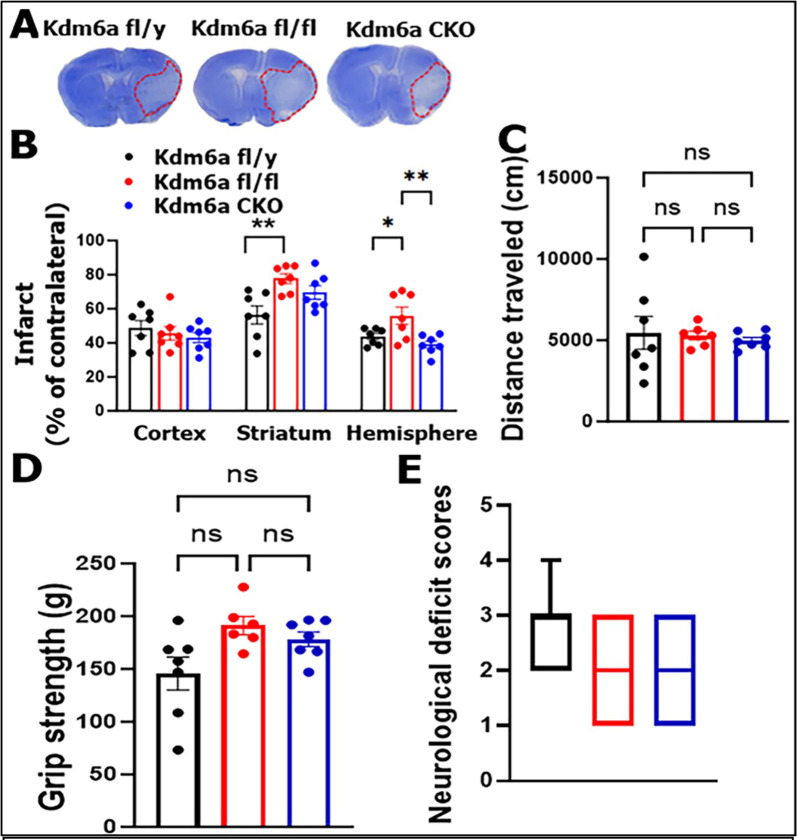
Stroke outcomes in *Kdm6a* fl/y, fl/fl and CKO mice. **(A)** Representative brain slices stained with Cresyl violet. **(B)** Quantification of infarct volumes in the ipsilateral hemisphere. (**C**) Distance traveled in open field test. (**D**) Grip strength test. **(E)** Neurological deficits scores (NDS). n = 6–7 per group. 2-way ANOVA with Tukey’s multiple comparison test. *p < 0.05, **p< 0.005.

We next evaluated stroke outcomes in *Kdm6a*^fl/fl^, fl/y, and CKO aged mice by examining infarct volumes and a battery of neurobehavior tests, three days after MCAO. We found that the striatal infarct in *Kdm6a*^*fl*/fl^ mice were significantly larger vs. fl/y mice. In addition, the fl/fl mice had significantly larger infarct in total ipsilateral hemisphere than fl/y or CKO mice (Fig. 7A, B). However, there were no significant differences between the strains in distance travelled (open field test) (Fig. 7C), grip strength (Fig. 7D) and NDS (Fig. 7E) at the acute timepoint (3d) after stroke.

### Kdm5c’s effect on IRF5 transcription does not contribute to stroke outcomes

**Figure 8:**
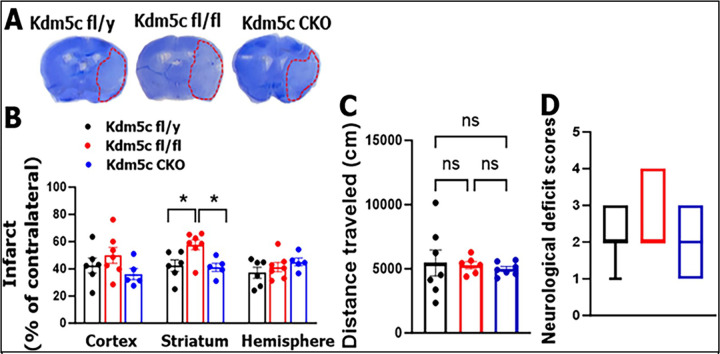
Stroke outcomes in *Kdm5c* fl/y, fl/fl and CKO mice. **(A)** Representative brain slices stained with Cresyl violet. **(B)** Quantification of infarct volumes in the ipsilateral hemisphere. (**C**) Distance traveled in open field test. (**D**) Neurological deficits scores (NDS). n = 6–7 per group for CV staining, distant traveled and NDS. 2-way ANOVA with Tukey’s multiple comparison test. *p < 0.05.

We also examined the effect of *Kdm5c’s* signaling on stroke outcomes, and found the similar results as that of *Kdm6a*. *Kdm5c* ^fl/fl^ mice exhibited significantly larger infarcts in the striatum compared to fl/y or CKO mice after 3d of stroke, but no differences were observed in neurobehavior deficits (Fig. 8A–D). IRF5 signaling is detrimental in stroke [21, 22], but in Fig. 3 we found two alleles of *Kdm5c* suppressed IRF5 transcription. Our data (Fig. 3 & Fig. 8) suggest *Kdm5c’s* detrimental effect on stroke injury is independent of IRF5 signaling.

## Discussion

It is well known that some X chromosome genes escape XCI [14, 15, 41, 42], leading to gene dosage imbalance between males and females [43, 44], which could impact post-stroke inflammation and outcomes once a stroke occurs. The present study focused on two X chromosome escapee genes, *Kdm6a* and *Kdm5c*, and investigated their epigenetic modulation of IRF5/IRF4 via demethylation of H3K27Me3/H3K4Me3 in aged microglia after stroke. IRF5-IRF4 regulatory axis has been previously found to be the determinant pathway that regulates microglial pro-/anti-inflammatory responses [21, 22, 28, 45, 46], and is critical in mediating stroke injury. The current data showed that Kdm6a and Kdm5c signaling both impact on one end of the axis, i.e. IRF5, but in an opposite pattern. Two alleles of *Kdm6a* led to active transcription of *IRF5*; whereas two alleles of *Kdm5c* caused suppressive transcription of the pro-inflammatory factor. Two alleles of either *Kdm6a* or *Kdm5c* in microglia induced exacerbated pro-inflammatory responses after stroke, which led to worsened stroke injury. The different effect of the two Kdms on *IRF5* transcripti on suggests that the two X escapee genes impact on stroke outcomes in the aged via different pathways.

Stroke is a sexually dimorphic disease [47, 48]; ischemic stroke sensitivity is mediated primarily by gonadal hormones in young population [49, 50] and by sex chromosomal complement in the aged [23, 51]. The contribution of the second X chromosome to stroke sensitivity in the aged has been observed in our previous study, with the two XCI escapee genes (*Kdm6a/Kdm5c*) involved [24]. The double expression of the two alleles of *Kdm6a/Kdm5c* due to the escape has been found also implicated in sex differences in cardiac infarction and adiposity [52, 53]. The current study utilized three animal models with different allele numbers of active *Kdm6a* or *Kdm5c*, and demonstrated the detrimental effects of both X escapee genes on stroke injury. Since the *Kdm* CKO female mice only has one allele of *Kdm6a* or *Kdm5c* and without Y chromosome, the comparison between CKO and *Kdm*^fl/fl^ females is exclusively reflective of the effect of X chromosome dosage but none of Y effect. Therefore, the current data convincingly indicate that the escape of *Kdm6a* or *Kdm5c* plays a detrimental role in post-stroke inflammation and stroke injury, and support the rational that the Y chromosome has limited effect on the stroke sensitivity [23].

The current study focused on the effect of *Kdm6a/5c* escape from XCI in aged microglia on stroke, as microglia play important roles in initiating and perpetuating post-stroke neuroinflammation. The inducible CKO model utilized in the study makes it feasible to investigate gene escape in microglia specifically. Although CX3CR1-CreER system targets both microglia and infiltrating monocytes in the ischemic brain, we did not perform experiments until 6 weeks after TMX induction so that the microglia can be the sole target. Infiltrating monocytes have gone through ‘turnover”[54, 55] and no longer bear the TMX induced gene knockout after 6 weeks of TMX induction; whereas microglia still have the KO due to their longevity [56]. Gene escape from XCI is random, and has tissue and cell variability [57, 58]. In addition, gene escape from XCI may be affected by various biological homeostasis changes including aging and stroke injury. XCI becomes unstable with age, which is a frequently proposed explanation for the phenotype spectrum of disease in females [42, 59, 60], suggesting some X-linked genes escape more easily with aging. Our previous study has found *Kdm6a/5c* were significantly higher expressed in sorted aged female vs. male microglia from naïve mice, and the sex difference was lost when evaluated in whole brain tissue in sham mice but present in brain tissue homogenates after stroke [24]. These data suggest that *Kdm6a/5c* escape from XCI has cell variability, and is sensitive to stroke stimulus.

Epigenetic regulation of genes has been widely studied including DNA methylation [61], histone [62] and non-coding RNAs [63] modifications, with growing interest in exploring the related regulatory mechanisms underlying neuroinflammation in stroke [11, 64, 65]. Techniques such as chromatin immunoprecipitation (ChIP) [62, 66], and CUT&RUN [67–69] have accelerated the advance of epigenetic studies, by elucidating gene-protein interactions and the downstream targets. Epigenetics involves histones which serve as “gatekeepers” to modulate DNA replication/transcription and gene expression [70]. Kdm6a and Kdm5c are demethylases for H3K27Me3 [71] and H3K4Me3 [72], and the demethylation of the two histones induces active and a transcriptive effect on gene transcription [73–76], respectively. Recently we have demonstrated by ChIP that the inflammatory transcription factors, IRF 5/4, bind to H3K27Me3 or H3K4Me3, suggesting the two IRFs are subjected to the epigenetic modulation of the histones [24]. Histones contain five components: H1, H2A, H2B, H3, and H4 [12], and undergo post-translational modifications of the N-terminal tail by acetylation, methylation, phosphorylation, ubiquitination, demethylation, and lactylation [77–80]. The modifications of histone tails affect the interaction of histones and DNA, and alter the structure and stability of chromatin [81], and regulate the gene transcription through modulating the affinity of transcription factors and structural gene promoters [14]. X chromosome-linked genes have been shown to play important roles in epigenetic modification of genes related to post-stroke inflammation [23, 82]. Our data show that kdm6a/5c both regulate *IRF5* transcription as in (Fig. 2&3), however in an opposite pattern (active vs. suppressive) through different histone demethylation, reflecting the complex nature of histone chromatin accessibility to transcriptional elements of the *IRF5* gene after stroke. Epigenetic mechanisms after stroke are critical in the molecular pathophysiology of the disease, and are potential therapeutic targets [64, 83] to salvage the hypoperfused ischemic penumbra that has not yet evolved into infarcted tissue [84]. The present study provided potential epigenetic avenues to target XCI escapee genes to regulate the expression of the pro-inflammatory transcription factor IRF5.

IRF5 is a well-established pro-inflammatory transcription factor responsible for mediating microglial production of inflammatory cytokines [21]. Of note, our data demonstrated that Kdm6a and Kdm5c signaling have opposite effects on IRF5 transcription (Figs. 2&3). However, the escape of both *Kdms* from XCI has pro-inflammatory effects including promoting microglial pro-inflammatory response (Fig. 4), increasing plasma/brain levels of pro-inflammatory cytokines (Fig. 5&6), and both led to exacerbated stroke injury (Figs. 7&8). The active effect of Kdm6a on *IRF5* transcription is logic to the downstream pro-inflammatory response and worsened stroke injury, but the suppressive effect of Kdm5c on *IRF5* seems irrelevant to the downstream outcomes. Different *Kdm* family proteins finetune the switch of gene expression by manipulating active or repressive histone methylation markers, thus participating in various links of immune cells and inflammatory activities [85, 86]. *Kdm6a* is a demethylase for H3K27Me3 [87], whereas *Kdm5c* is responsible for demethylation of H3K4Me3 [88]. Our data are consistent with this as H3K4-IRF5 axis was not affected by Kdm6a (Fig. 2D–F) and K3K27-IRF5 not changed by *Kdm5c* (**Suppl. 2 A-C**). The specific histone target for the two Kdms might be the reason why they have different effect on *IRF5* transcription. It is likely that *Kdm5*c suppresses transcription of some anti-inflammatory genes to confer detrimental effects on neuroinflammation.

The current study has some caveats that we should keep in mind when interpreting the data. We examined the Kdm-histone-IRF axis in aged microglia only at the acute phase of stroke (3 days after MCAO), and did not include a chronic stage cohort study which is still on-going (years of work). However, our acute study has already elucidated the mechanistic link between Kdm6a/5c and post-stroke inflammation, which will be further confirmed in the following experiments. Another caveat of the study is that we did not examine Kdm-IRF5 signaling in infiltrating monocytes. It has been reported [89] that demethylation of H3K27Me3 by Kdm6a markedly increased IL-1β expression through a Caspase-1 pathway in macrophages. The infiltrating monocytes in the ischemic brain also express IRF5 [45, 90, 91]. Nevertheless, our previous study has already suggested that the central (microglia) IRF signaling is more important than the IRFs expressed on peripheral immune cells in post-stroke inflammation [92].

**Figure 9:**
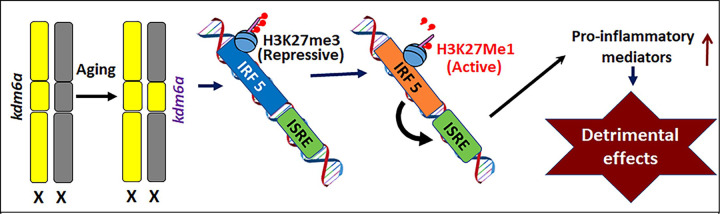
Mechanistic diagram. *Kdm6a* escapes from XCI in aged microglia and demethylates H3K27Me3 (suppressive form) to H3K27Mel (active). H3K27Mel then binds IRF5 gene and activates IRF5 transcription, leading to up-regulation of the expression of pro-inflammatory mediators.

In summary, the present study investigated the demethylating effects of Kdm6a/5c on H3K27Me3/H3K4Me3-IRF5/4 signaling in microglia, and assessed their impact on stroke outcomes in aged mice. Our findings reveal that the escape of microglial *Kdm6a/5c* from XCI exacerbates post-stroke inflammation and worsens outcomes. IRF5 signaling plays a critical role in mediating the deleterious effect of *Kdm6a* (Fig. 9); whereas *Kdm5c’s* effect is independent of IRF5. The epigenetic modification of histones by X escapee genes is a novel mechanism in inducing sex differences in stroke among the elderly, highlighting new, sex-specific therapeutic targets for this devastating disease.

## Data Availability

The datasets used and/or analyzed in the present study are available from the corresponding author upon reasonable request.

## Abbreviations

ATCC: American Type Culture Collection
BCA: Bicinchoninic acid assay
ChIP: Chromatin immunoprecipitation
CKO: Conditional knock out
CUT&RUN: Cleavage Under Targets & Release Using Nuclease
ELISA: Enzyme-Linked Immunosorbent Assay
fl/fl: flox/flox
H3k27Me3: trimethylation of histone H3 at lysine 27
H3k27Me1: monomethylation of histone H3 at lysine 27
H3k4Me3: trimethylation of Histone H3 at Lysine 4
H3k4Me1: monomethylation of Histone H3 at Lysine 4
ISRE: Interferon stimulatory regulatory element
IRF5: Interferon regulatory factor 5
IRF4: Interferon regulatory factor 4
*Kdm6a*: Lysine-specific demethylase 6A
*Kdm5c*: Lysine demethylase 5C
MCAO: Middle cerebral artery occlusion
MFI: Mean fluorescence intensity
RPMI: Roswell Park Memorial Institute
RT: Room temperature
USP9X: Ubiquitin specific peptidase 9 X-linked
XCI: X chromosome inactivation
